# Remote Testing of the Familiar Word Effect With Non-dialectal and Dialectal German-Learning 1–2-Year-Olds

**DOI:** 10.3389/fpsyg.2021.714363

**Published:** 2021-12-02

**Authors:** Bettina Braun, Nathalie Czeke, Jasmin Rimpler, Claus Zinn, Jonas Probst, Bastian Goldlücke, Julia Kretschmer, Katharina Zahner-Ritter

**Affiliations:** ^1^Department of Linguistics, University of Konstanz, Konstanz, Germany; ^2^School of Education, University of Leeds, Leeds, United Kingdom; ^3^Institute of Phonetics and Speech Processing, University of Munich, Munich, Germany; ^4^Department of Linguistics, University of Tübingen, Tübingen, Germany; ^5^Department of Computer and Information Science, University of Konstanz, Konstanz, Germany; ^6^Department of Phonetics, University of Trier, Trier, Germany

**Keywords:** familiar word effect, remote testing, iPad App, word representation, children, German, regional variation, dialect

## Abstract

Variability is pervasive in spoken language, in particular if one is exposed to two varieties of the same language (e.g., the standard variety and a dialect). Unlike in bilingual settings, standard and dialectal forms are often phonologically related, increasing the variability in word forms (e.g., German *Fuß* “foot” is produced as [fu
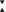
s] in Standard German and as [f
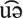
s] in the Alemannic dialect). We investigate whether dialectal variability in children’s input affects their ability to recognize words in Standard German, testing non-dialectal vs. dialectal children. Non-dialectal children, who typically grow up in urban areas, mostly hear Standard German forms, and hence encounter little segmental variability in their input. Dialectal children in turn, who typically grow up in rural areas, hear both Standard German and dialectal forms, and are hence exposed to a large amount of variability in their input. We employ the familiar word paradigm for German children aged 12–18 months. Since dialectal children from rural areas are hard to recruit for laboratory studies, we programmed an App that allows all parents to test their children at home. Looking times to familiar vs. non-familiar words were analyzed using a semi-automatic procedure based on neural networks. Our results replicate the familiarity preference for non-dialectal German 12–18-month-old children (longer looking times to familiar words than vs. non-familiar words). Non-dialectal children in the same age range, on the other hand, showed a novelty preference. One explanation for the novelty preference in dialectal children may be more mature linguistic processing, caused by more variability of word forms in the input. This linguistic maturation hypothesis is addressed in Experiment 2, in which we tested older children (18–24-month-olds). These children, who are not exposed to dialectal forms, also showed a novelty preference. Taken together, our findings show that both dialectal and non-dialectal German children recognized the familiar Standard German word forms, but their looking pattern differed as a function of the variability in the input. Frequent exposure to both dialectal and Standard German word forms may hence have affected the nature of (prelexical and/or) lexical representations, leading to more mature processing capacities.

## Introduction

Testing children’s word recognition has become an important cornerstone in developing models of lexical representation during the first two years of life. Developmental psychologists have so far paid little attention to how long-term exposure to more than one variety of a language affects children’s word recognition abilities. Hence, the nature of early lexical representations in children who grow up with two varieties of a language (e.g., Standard German and a dialectal variant, henceforth “dialectal” children) remains largely unclear. The main aim of the present study is to compare the recognition of Standard German word forms in dialectal and non-dialectal German children. We present a method to reach these dialectal children, using an App for iPads for remote testing of word form recognition.

We tested children’s word form recognition using the familiar word paradigm. In this paradigm, children from around 11 months onward have been shown to attend longer to familiar word lists than to unfamiliar or nonce-word lists, hence showing a preference for known words (familiarity preference), which is taken to reflect successful word form recognition (e.g., Hallé and Boysson-Bardies, 1994; Vihman et al., 2004; Carbajal et al., 2021 for a meta study). Children are commonly tested in the lab in a head-turn preference paradigm, HPP (Hallé and Boysson-Bardies, 1994; Vihman et al., 2004; van Heugten and Johnson, 2014) or a visual-fixation paradigm (Best et al., 2009), both of which employ child-controlled stimulus presentation. We chose the familiar word paradigm for two reasons: First, it focuses on the processing of word forms, which may differ for dialectal children who grow up with two varieties of the same language (Standard German and a dialect). Second, the familiar word paradigm is robust (Carbajal et al., 2021), which makes it suitable for replication outside the lab using an App in a more natural but potentially also more distracting environment.

In the present paper, we study whether exposure to a dialect in addition to the Standard affects German-learning children’s word recognition abilities. In Experiment 1, we compare two groups of children: (a) 12–18-month-olds who grow up with Standard German only (“non-dialectal children”) and (b) 12–18-month-olds who grow up with Standard German and an additional German variety (“dialectal children”). Both groups are tested outside the lab using an experiment-controlled visual fixation procedure implemented in an App. In Experiment 2, we included older non-dialectal children (18–24 months of age) to test one hypothesis that may explain the different patterns of results for dialectal and non-dialectal children in Experiment 1. In the following, we will give a brief overview of dialectal variation in Germany and focus on the coding of dialectal input in more detail (see section “Dialectal Variation in Germany and the Coding of Dialectal Input”), before we move on to review the literature on early word form recognition (see section “Word Form Recognition in Light of Dialectal Exposure”).

## Background

### Dialectal Variation in Germany and the Coding of Dialectal Input

**FIGURE 1:**
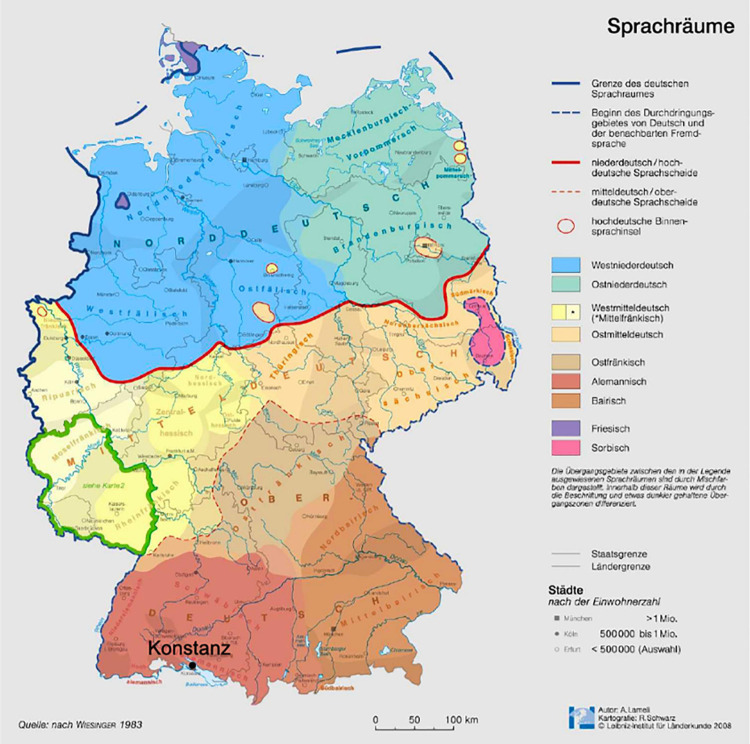
Map of German dialects (http://aktuell.nationalatlas.de/Dialektraeume.9_08-2008.0.html/). The relevant dialect areas in the South of Germany translate as follows: *Schwäbisch* “Swabian,” *Mittelalemannisch* “Alemannic,” *Mittelbayrisch* “Middle Bavarian.” Reprinted with permission of Alfred Lameli.

Germany is historically divided into different dialectal areas, see Figure 1. In their original form, these dialects are difficult to decode for outsiders because they do not only differ in phonology and phonetics, but also in morphology and syntax (Bayer, 1984; Siebenhaar and Wyler, 1997; Brandner and Saltzman, 2009; Grewendorf and Weiß, 2014). We focus on the dialectal areas in Southern Germany (Alemannic and Bavarian, red and orange in Figure 1), as most of the dialectal children tested in our study grow up in these regions.

In addition to grammatical and morphological differences, there is a large range of phonological and phonetic differences between dialectal word forms in Alemannic and Bavarian as compared to Standard German forms (Siebenhaar and Wyler, 1997; Munske, 2015). A comprehensive introduction to dialectal phonology is beyond the scope of this paper; we will focus on main differences here (see Table 1, for examples). Consonantal differences include lenition of plosives (1), place of articulation of the fricative /s/ (2) in Alemannic, vocalization of coda-liquids (3) in Bavarian, devoicing of word-initial [z] (4), vocalic differences include schwa-elision (5), and diphthongization (6, 7), see Table 1.

**TABLE 1:** Example of differences between Standard German, Alemannic, and Middle Bavarian.

		Standard German	Alemannic	Bavarian	English translation
1	[t^h^]	[ 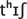 ] (Tisch)	[ 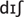 ]	[ 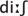 ]	table
2	[s]	[ 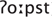 ] (Obst)	[ 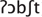 ]	[ 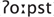 ]	fruits
3	[l]	[ 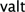 ] (Wald)	[ 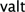 ]	[ 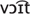 ]	forest
4	[z]	[ 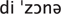 ] (die Sonne)	[ 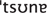 ]	[ 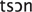 ]	sun
5	[e]	[ 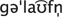 ] (gelaufen)	[ 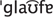 ]	[ 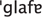 ]	ran
6	[u 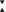 ]	[ 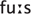 ] (Fuß)	[ 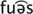 ]	[ 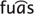 ]	foot
7	[y 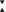 ]	[ 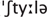 ] (Stühle)	[ 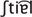 ]	[ 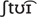 ]	chairs

There is substantial variation in the (proportion of) usage of dialectal forms in Southern Germany (Schwarz, 2014). In more rural areas of Germany, dialect is frequently used for daily communication between locals (Schwarz, 2014). Standard German, the variety present on national TV and in schools, in turn, is spoken when reading to children, in audio books, on radio and TV, and in more formal situations (at the pharmacy, the doctor’s etc.). Since Standard German is used in the educational context (school, university), parents may have an incentive to introduce this variety to their children from early on. In any case, Standard German word forms regularly occur in addition to dialectal word forms (around 50% of spontaneous speech tokens in a 2,000 word corpus are dialectal forms in Schwarz, 2014, p. 77), and one and the same caregiver may even switch between dialectal and Standard German forms. The usage of dialectal forms is gradient and a higher proportion of dialectal word forms increases the perceived strength of a speaker’s dialect. In contrast, in more urban areas, dialect is (and has increasingly become) less frequent (Schwarz, 2014), probably because the population is more heterogenous, making Standard German the most comprehensible style. Most of our non-dialectal children came from Konstanz, a university city of around 85,000 inhabitants at the lake of Konstanz (see Figure 1). The proportion of dialectal forms is substantially smaller in Konstanz compared to more rural areas (Schwarz, 2014, p. 77), with Standard German prevailing in most contexts.

Children growing up in Southern Germany hence differ in the amount of exposure to dialectal word forms they receive. In a recent study on the phonological variability in infant-directed speech, Zahner et al. (2021) showed that around one third of the word forms of dialectal parents contained a dialectal feature (i.e., a segmental deviation from the Standard form), while two thirds of the word forms were (congruent with) Standard German. Children growing up in Konstanz, on the other hand, were only exposed to around 5% of dialectal forms, with a huge majority of word forms being Standard German. These proportions reported in Zahner et al. (2021) were constant across different recording settings (naturalistic home recordings vs. lab-like picture book descriptions). The amount of dialectal exposure a child receives thus seems to depend on the region (more rural or urban), but also on the parental attitude toward their usage of dialect (cf. personal communication with families in our lab in Konstanz). Exclusively taking into account a child’s residence is therefore an insufficient proxy for the amount of dialectal exposure it receives. Complementary perceptual judgments of dialectal input on the other hand seem a valid tool for the classification of a child’s exposure to dialectal input (in addition to the Standard): Researchers have most often used rating scales with four categories (van Bezooijen and van Hout, 1985; Stölten and Engstrand, 2003; Floccia et al., 2009), but there are also studies that employed seven categories (Grondelaers et al., 2015), magnitude estimation (Brennan et al., 1975), or handgrip force (Brennan et al., 1975). For the purpose of this paper, children were divided into groups of dialectal vs. non-dialectal children according to the perceived dialectal strength of parental productions, which, as we will show, are correlated with the proportion of dialectal word forms that children are exposed to (see section “Participants”). The question emerging from the different amount of exposure to dialectal forms is whether long-term exposure to word forms in more than one variety of a language affects children’s word recognition abilities. The present study is designed to fill this gap. We will now turn to previous findings on word form recognition, particularly focusing on studies that test infants with exposure to more than one variety.

### Word Form Recognition in Light of Dialectal Exposure

On their way toward learning words and building a vocabulary, one of the tasks children need to master is to acquire and refine word form representations (Westermann and Mani, 2018, for an overview). Children’s ability to recognize word forms is commonly tested in the familiar word paradigm in which they are presented with two types of words: (familiar) words vs. nonce-words/rare words. As mentioned above, successful recognition of words typically surfaces in a preference for words over less familiar or nonce words (Carbajal et al., 2021). Children from a large number of different languages, including British and American English, Dutch, French, Spanish, Italian, and Japanese have been shown to recognize words from the end of the first year of life onward (26 experiments in Carbajal et al., 2021 tested children between 10 and 12 months of age), hence having started to develop lexical representations. The familiar word effect is influenced, among others, by children’s age (stronger familiarity preference with increasing age), native language (stronger familiarity preference in Romance compared to Germanic languages), and their degree of word familiarity within real word lists (stronger familiarity preference when more familiar words were used). Under the assumption that the familiar word effect extends to German (a language not yet tested in this paradigm) and remote testing (previous studies were conducted in the lab), we predict a replication of the familiar word effect for non-dialectal German children aged 12–18-months (hypothesis H1). These children grow up with Standard German only which is why the presented Standard German word forms are assumed to be highly familiar to them. We explicitly included children older than 1 year of age (and hence older than children in most of the previous studies, cf. Carbajal et al., 2021) due to reasons of comparison between dialectal and non-dialectal children (for whom successful recognition of Standard forms might be observed later, see below). Another reason for testing 12–18-month-olds was that testing conditions outside the lab are different from typical laboratory settings, with less experimental control potentially leading to a reduction of the effect. This older age range might hence result in a more robust recognition effect.

In spoken communication, word forms are essentially variable, and children have to learn to recognize them in different (more or less variable) contexts (cf. White, 2018 for an overview). Indeed, it has been shown that children find it hard to recognize words when they are spoken with an *unfamiliar* accent. (Monolingual) children succeed in this task only by the end of the second year of life (Best et al., 2009; van Heugten and Johnson, 2014; van Heugten et al., 2018), suggesting very rigid early lexical representations that do not allow for deviations from the form children are familiar with [but see Schmale et al. (2010) showing successful word recognition from around the first year of life in a different paradigm]. The situation, i.e., the mental representation of words, is probably different for children who grow up with two varieties of one language at a time. So far, however, we know very little about how long-term exposure to two varieties of a language affects the ability to recognize words, and the nature of early representations in these “bi-varietal” or dialectal children. For instance, children growing up in rural areas of Southern Germany are exposed to both Standard and dialectal forms (see section “Dialectal Variation in Germany and the Coding of Dialectal Input”), and regularly encounter both [fu
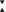
s] (Standard for “foot”) and [f
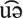
s] (Alemannic for “foot”) in their input. Conceivably, the exposure to two varieties at a time affects how words are represented (see below).

Taken together, bi-varietal or dialectal upbringing leads to more variability in the input, but at the same time, also leaves the child with less input in either of the two varieties. Models of infant word recognition would generally predict that increased variability in the input is beneficial for the refinement of word forms and therefore facilitates the recognition of novel tokens (Johnson, 2016; see van Heugten and Johnson, 2017 for discussion). In this regard, speaker variability has indeed proven to be beneficial in word recognition and word learning (Singh, 2008; Rost and McMurray, 2009; Höhle et al., 2020). Little is known, however, about the effect of dialectal/varietal variability on word form recognition. There is one study by van Heugten and Johnson (2017) that investigated whether exposure to multiple accents affects the recognition of word forms. Specifically, the authors compared looking times to word lists containing familiar English words and nonce-word lists in children with low variability in the input (mainly exposed to Canadian English input, i.e., only one variety of English) vs. with high variability in the input (with around one third of exposure to Canadian English and two thirds of exposure to a different type of non-Canadian English, either another native English variety or foreign-accented English). Their results showed that while 12.5-month-old children from the low variability group successfully recognized Canadian English words, the high variability group only succeeded in this task at the age of 18 months. These findings hence suggest that exposure to multiple accents might in fact delay the familiar word effect rather than leading to beneficial processing. Based on these findings, we tentatively assume that the familiar word effect may surface later in our dialectal group as compared to their non-dialectal peers (hypothesis 2, H2). It needs to be mentioned though that the group of children tested in van Heugten and Johnson (2017) is more heterogenous compared to our group [all of our children are exposed to a native, Southern German dialect while children in van Heugten and Johnson (2017) are exposed to different types of native and non-native varieties], which might reduce direct comparability of the two studies.

While studies employing the familiar word paradigm may trace the development of word recognition abilities in different groups of children (e.g., mono- vs. bi-varietal children), they cannot directly answer questions on the nature of lexical representations. This, however, is particularly relevant for children who grow up with two varieties. Bi-varietal or dialectal children may initially store (a) the form of one variety only (and thus only recognize the word forms of one variety, cf. Floccia et al., 2012), (b) the forms of both varieties (thus recognizing word forms of both varieties, cf. van der Feest and Johnson, 2016), or (c) develop underspecified representations (thus also accepting word forms with unattested phonological alternations, cf. Durrant et al., 2015). To answer such specific questions on the nature of lexical representations within the framework of the familiar word paradigm, one would need lists of words and corresponding nonce-words that are segmentally similar (e.g., all starting with the same consonant or having the same vowel) so that the reaction to a specific deviance can be tested. In this paper, we take a first step in this direction and use sets of words that share the same stressed vowel (both in the word and nonce-word tokens). For this purpose, we created two different word lists, one consisting of segmentally similar words that all contain the vowel [u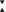] as stressed vowel (u-only condition), and another consisting of segmentally varied words that contain mixed vowels in stressed position (u-varied condition). If the familiar word effect is comparable for mixed and segmentally similar word lists, future research could test the above-mentioned types of representations within this paradigm. For now, a successful recognition of Standard word forms in our dialectal group (which is expected to emerge later than in the monolingual group, cf. H2), would sustain the possibility of underspecified representations or double storage of forms in both varieties in dialectal children.

The remainder of this paper is structured as follows: In section “General Information on the App”, we first introduce the App that was developed and used to test a wide range of children in their home environments, and then describe the manual and semi-automatic coding that was used to analyze looking behavior. In section “Experiment 1: Word Form Recognition in 12–18-Month-Old Children,” we test the familiar word effect with German children between 12 and 18 months of age (Experiment 1). We manipulated dialectal input (between-subjects) as well as the nature of the materials (between-subjects) using different word lists (u-varied vs. u-only). Section “Experiment 2: Non-dialectal 18–24-Month-Old Children” tests non-dialectal children between 18 and 24 months of age (Experiment 2). In section “General Discussion,” we discuss the looking behavior and possible interpretations for word representations in dialectal and non-dialectal children. The same section concludes with discussing the applicability and limitations of the App used to study the familiar word effect with a more (linguistically and demographically) diverse population.

## General Information on the App

The App was developed by the fourth author (CZ). It is freely available in the App store^1^.

### Introduction of the App

**FIGURE 2:**
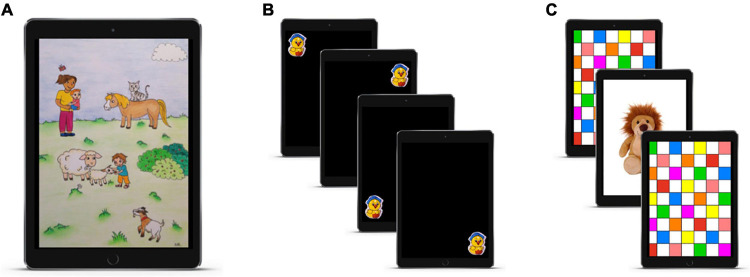
Illustration of the individual experimental steps as displayed in the App, including **(A)** the production phase, **(B)** the calibration phase, and **(C)** an example of two experimental trials separated by an attention getter.

In brief, a video introduces caregivers (mostly the mother) to the general procedure of the App, before they give consent and fill in a background questionnaire. In particular, we asked about the language(s) and dialect(s) the child is exposed to, and about the highest education of both caregivers as a proxy for socioeconomic status (Hoff et al., 2002; Bornstein et al., 2003; Ensminger and Forthergill, 2003; and references therein; Noble et al., 2005; Sirin, 2005). Furthermore, we asked whether the child is typically developed and whether there are any impairments in vision or hearing. The first phase of the experiment is a short production phase in which a caregiver describes a colorful picture to the child. The picture displays different people and animals, see Figure 2A. Parental speech input is used to judge the amount of dialectal variation a child receives (see section “Participants” for more details). The word recognition experiment itself starts with a calibration phase (an animated duck which appears in four corners of the screen) in order to establish reference points for manual coding (whether or not the child looks on the iPad or beyond its borders), see Figure 2B. The calibration phase is followed by the experimental trials of the word recognition experiment (see section “Procedure” for details). After the word recognition experiment, the caregiver is asked whether the other caregiver would like to describe the picture to the child again, whether there was any distraction during the word recognition experiment, and whether they would like to take part in a raffle. The data are then encrypted and securely transferred onto a password-protected university server for subsequent analysis.

### Manual and Semi-Automatic Analysis of Looking Behavior

All eight experimental trials of each child were screened by one author (JK) to check the number of trials that a child completed. We included children who completed a minimum of six trials. To train the classifier for automatic coding, we selected two of the eight trial videos that differed most strongly in the child’s orientation and/or movement. Looking behavior in those trial videos was coded manually frame-by-frame in ELAN (Brugman and Russel, 2004; ELAN, 2020), an annotation tool for video (and audio) recordings, as “look”, “no-look”, or “undecided”. All annotators were trained and received individual feedback on a set of videos that had previously been coded by two of the authors, both experienced in video annotation with ELAN (NC and KZ-R). Annotators received individual feedback on their annotations and training was completed once (a) the annotators felt confident in the coding process and (b) their annotations repeatedly did not differ more than ± one frame from the boundaries in the annotation by the two authors that was used for baseline comparison, respectively. This was the case after no more than ten videos.

In total, four coders annotated the children’s looking behavior. To determine the interrater agreement, we analyzed the coding of a coder pair frame-by-frame for a total of 24 videos (2 trials with a duration of 15 s from 12 children). The average agreement was 88.9%, Cohen’s kappa 0.77, which is substantial (Landis and Koch, 1977), cf. Table 2.

**TABLE 2:** Pairwise interrater agreement for the pairs of coders for look/no-look.

Annotator pair	Agreement
Annotator1-Annotator2	92.3%
Annotator2-Annotator3	86.6%
Annotator2-Annotator4	81.3%
Annotator3-Annotator4	95.4%

Using the manually annotated trials, a semi-automatic annotator was trained to process the remaining videos. The semi-automatic annotation is a two-step process: In the first step, parameters are extracted from the videos, using face and landmark detection software from 4dface^2^. In the second step, each frame is classified using a Long-term Recurrent Convolutional Network (cf. Donahue et al., 2015 for successful application in human action classification).

For parameter extraction, all faces which are visible in the video are detected using the deep learning-based face detector from 4dface and the child’s face is selected either automatically based on position (lower on screen, more centered) or manually in ambiguous situations (e.g., presence of a sibling). Then, the face is tracked over the duration of the video and the facial landmarks are localized. Seven parameters are calculated for each frame: the face’s orientation (pitch, yaw, and roll), the relative x and y position to the center of the image, the distance between the outer corners of the eyes and the distance between the chin and the center point between the eyes. Additionally, a cropped image of each eye is saved for each frame. For frames in which the face is not detected, placeholder values are saved instead.

**FIGURE 3:**
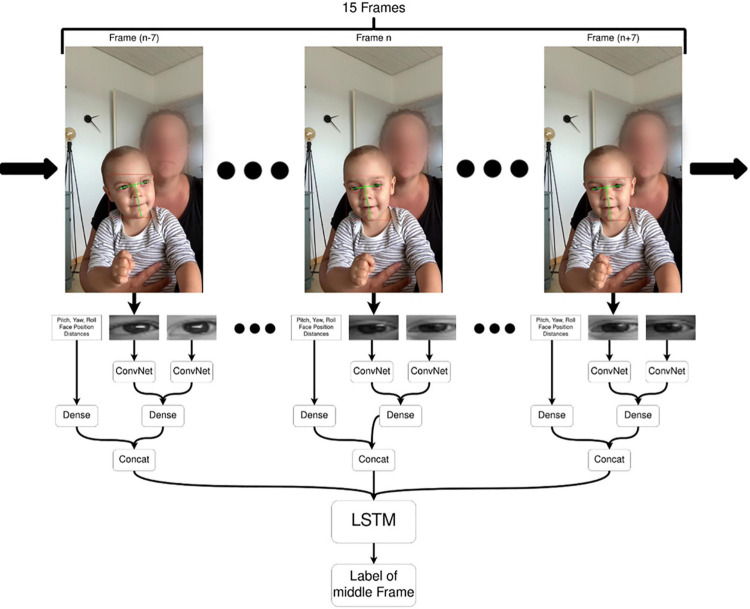
Overview of the automatic coding procedure.

For the classification step, we constructed and trained a Long-Term Recurrent Convolutional Network (Donahue et al., 2015), which combines the previously calculated numeric parameters with the eye images and returns a label for each frame. The LSTM is capable of incorporating temporal context to the classification, so the input to classify one frame is not only based on the parameters of the frame itself, but also on the seven frames before and after. This improves classification of frames in which the eyes are not visible because of occlusion, or frames in which the child is blinking. A simplified representation of the model can be seen in Figure 3. In the network, the eye images are fed through a Convolutional Neural Network (ConvNet), which learns to interpret the images and represents them in a dense layer. The dense layer is concatenated with the other numeric parameters, which are then fed into the LSTM. The LSTM assigns the label “look” or “no look” to classify the middle frame; the “undecided” category is ignored during training because it does not contain enough data points. To deal with outliers, the resulting series of frame classifications is smoothed. This corrects some erroneous classifications, which are mostly single frames that are classified with a different label than the ones surrounding it.

For training the LSTM, the manually annotated videos are split in a training (75%) and validation set (25%), making sure that at least one video of each child occurs in the training set. Ten videos are held back as a test set. The training set is augmented with a mirrored version of each video. Using drop-out and kernel regularization on the dense layers is essential to prevent over-fitting. The final model achieves an average agreement between the manual annotations and the semi-automatic annotations of 97% on the training set, 94% on the validation set and 93% on the test set. The average Cohen’s kappa between the manual annotations and the semi-automatic annotations is 0.83 on the test set.

## Experiment 1: Word Form Recognition in 12–18-Month-Old Children

Experiment 1 is a replication of the familiar word paradigm to test word form recognition of German non-dialectal and dialectal 12–18-month-olds, using the App.

### Methods

#### Participants

The grouping of children into a non-dialectal and a dialectal group was based on the parental recordings (gathered in the production phase, see Figure 2A). To this end, the input data were converted to the wav media format and each rated by four student assistants with respect to dialectal strength. The student assistants were all students of linguistics (at least in their 2nd year). There were two fixed sets of four student assistants. The student assistants were selected based on their place of origin in Germany to reduce effects of familiarity with some of the dialects. The coders rated the dialectal strength on a 4-point Likert scale. Perfectly Standard German stimuli were coded as 1, stimuli with a few slight dialectal features as 2, with more dialectal features as 3, and 4 was used for highly dialectal productions, see Supplementary Table 1 for examples of dialectal productions. We calculated Cronbach’s α as a measure of reliability (Cronbach, 1951); α was 0.94 for the first set of annotators and 0.92 for the second set. We used the mode (most frequent rating among the four coders) to group children into two dialect groups. We used a mode of 2 as the cutoff-value. Non-dialectal children had a mode of 1 or 2, dialectal children had a mode of 3 or 4. There were four critical ties (mode 2 vs. 3), which were resolved by soliciting an additional rating of a randomly selected rater from the other group.

To support the classification into dialectal and non-dialectal children, a random selection of picture descriptions (four from the non-dialectal and four from the dialectal group) were coded phonetically, following the procedure described in Zahner et al. (2021). More specifically, each word form was coded according to its segmental deviation from the expected Standard German form due to dialectal variation, e.g., [nø
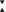
t] for [nIçt] *nicht* “not,” or due to general reduction processes that occur in connected speech, e.g., [nIç] for [nIçt] (Kohler, 1990). The proportion of dialectal word forms was 9% (SD = 2%) for the caregiver of the non-dialectal group and 34% (SD = 8%) for the dialectal group. The average dialectal strength (averaged over four rates) was highly correlated with the proportion of word forms that contain dialectal deviances (Spearman’s Rho = 0.88), see Supplementary Figure 1. This emphasizes the relation between the perception of dialectal strength and the frequency of occurrence of dialectal variants.

Forty-four children were included in the analyses of Experiment 1, 25 non-dialectal children (13 in the u-varied condition, 12 in the u-only condition) and 19 dialectal children (11 in the u-varied condition, 8 in the u-only condition). They were matched for gender and age (see Table 3 for details). Eleven of the children came from a Swabian area (Zip-Code 72, north of Konstanz), five from an Alemannic area (Zip-Code 78 and 79, Bodensee area) and three from Bavaria, south of Munich (Zip-Codes 82 and 83). Seven more children were tested, but not analyzed (4 non-dialectal and 3 dialectal) because the child did not pay attention (3 times), was not in the frame (2 times), was reported to be ill or impaired (1), or there was loud background noise (1). This resulted in a dropout rate of 13.7%.

**TABLE 3:** Overview of participant metadata.

	Non-dialectal children		Dialectal children	
	u-varied	u-only	u-varied	u-only
Gender (number of female/male)	6/7	7/5	5/6	3/5
Mean age (SD) in months	14.5 (1.5)	15.6 (1.9)	14.5 (2.1)	14.4 (1.9)
Mean dialect score	1.4 (0.4)	1.6 (0.4)	3.3 (0.5)	3.2 (0.3)
Highest education of caregiver 1 (university degree/vocational training/A-levels/O-levels)	9/2/2/0	9/2/1/0	4/5/1/1	3/2/3/0

#### Materials

##### Selection of Words and Nonce-Words

Eighteen frequent German words were selected, twelve words with the vowel /u/ and six words with mixed vowels [other long vowels (/a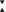/ and /i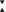/), other short vowels (/a/ and /œ/), and the diphthong /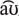/], see first two columns of Table 4.

**TABLE 4:** List of words and their IPA-transcription (first two columns) and the generated nonce-words (last two columns).

Word	IPA	dlexDB	WB 18 m/24 m	Nonce- word	IPA (Standard)
*Kuchen “cake”*	[ 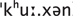 ]	0.98	0.16/0.66	*Buten*	[ 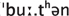 ]
*Fuß “foot”*	[ 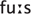 ]	2.07	*0.27/0.75*	*Stuch*	[ 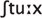 ]
*Kuh “cow”*	[ 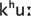 ]	1.18	0.22/0.8	*Fuh*	[ 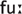 ]
*Stuhl “chair”*	[ 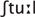 ]	1.70	0.19/0.71	*Guhm*	[ 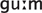 ]
*Schuh “shoe”*	[ 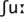 ]	1.45	0.45/0.78	*Kud*	[ 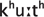 ]
*Buch “book”*	[ 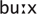 ]	2.34	0.39/0.86	*Zust*	[ 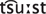 ]
*Blume “flower”*	[ 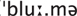 ]	1.65	0.28/0.72	*Bluche*	[ 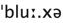 ]
*gut “good”*	[ 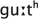 ]	3.05	0.12/0.47	*Suh*	[ 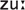 ]
*Bruder “brother”*	[ 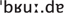 ]	2.02	0.02/0.25	*Schuser*	[ 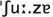 ]
*Husten/husten “(to) cough”*	[ 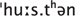 ]	1.28	–/–	*Bruchen*	[ 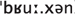 ]
*zu “to”*	[ 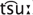 ]	3.96	0.28/0.57	*Hu*	[**hu** 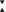 ]
*suchen “to search”*	[ 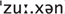 ]	2.37	–/–	*Kulen*	[ 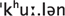 ]
*Hase “hare”*	[ 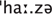 ]	0.91	0.27/0.73	*Kafe*	[ 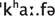 ]
*Baum “tree”*	[ 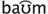 ]	1.87	0.26/0.73	*Haul*	[ 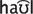 ]
*spielen “to play”*	[ 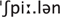 ]	2.32	0.13/0.58	*Biesen*	[ 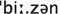 ]
*Katze “cat”*	[ 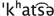 ]	1.29	0.26/0.76	*Lamme*	[ 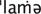 ]
*Ball “ball”*	[ 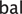 ]	1.20	0.76/0.95	*Spall*	[ 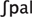 ]
*Löffel “spoon”*	[ 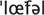 ]	1.05	0.19/0.71	*Bötzel*	[ 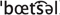 ]

*The first 12 pairs are used in the u-only condition, the first six and last six pairs in the u-varied condition. Segmental changes are highlighted in bold face. The middle columns give information on lexical frequency and production frequency at 18 months and at 24 months of age. Numbers in italics were only available for the plural form.*

All selected words had at least one consonant in the onset position of the first syllable. Nine of the words were monosyllabic (e.g., *Stuhl* [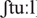], “chair”) and nine were disyllabic with a trochaic stress pattern (e.g., *Katze* [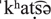], “cat”). They were all expected to be known by German 24-month-olds. They had a log-lemma frequency count higher than 0.9 from dlexDB (Heister et al., 2011) with an average of 1.85, see third column of Table 4. Furthermore, they are produced by at least a quarter of German 24-month-old children (average 69%), as indicated in Wordbank (Frank et al., 2017), an open database of children’s vocabulary growth. The Wordbank (WB) database^3^ collects MacArthur-Bates Communicative Development Inventory (MB-CDI) data in many different languages, i.e., information from parent-report questionnaires on children’s vocabulary growth (Szagun et al., 2009). The German data set consists of 1,181 children aged between 18 and 30 months. The retrieved WB data (fourth column of Table 4) shows the proportion of German children producing an item at a specific age. The words in the u-only and u-varied lists did not differ from each other in terms of frequency and word bank data (see Supplementary Table 2).

Nonce-words were constructed in the following way: For disyllabic words, we exchanged the onset consonants of the syllables of each word with those of another word (e.g., [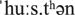] → [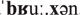]). For monosyllabic words, we exchanged onset and coda consonants between word pairs ([b
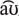
m] → [**h**
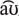
**l**]). Each word formed a pair with its newly constructed nonce-word, see Table 4, last two columns. This procedure ensured that the pairs of words and nonce-words were comparably complex regarding consonant clusters and syllable structure. Consonants were exchanged because they strongly affect word recognition (Poltrock and Nazzi, 2015). One important criterion for the nonce-word generation was that phonotactic probabilities were matched for words and nonce-words (Vitevitch and Luce, 2004, p. 481). Similar to the web-based Phonotactic Probability Calculator for English^4^ [used, e.g., in van Heugten et al. (2018)], we extracted positional segment frequencies and position-specific biphone frequencies for each (nonce-) word from the wordform dictionary of the CELEX lexical database (Baayen et al., 1995) using a self-programmed Python script. Table 5 shows that the mean phonotactic probabilities are matched at the segment and biphone level^5^.

**TABLE 5:** Mean phonotactic probabilities (and standard deviations) of words and nonce-words.

	u-only		u-varied	
	Words	Nonce-words	Words	Nonce-words
Segments	1.22 (0.13)	1.22 (0.13)	1.20 (0.09)	1.22 (0.10)
Biphones	1.02 (0.03)	1.03 (0.03)	1.02 (0.02)	1.02 (0.02)

##### Acoustic analyses

A 26-year-old female native speaker of Standard German from the Southwest of Germany (Baden-Wuerttemberg) recorded the thirty-six experimental items in isolation (18 words and 18 nonce-words). She was instructed to produce them as if naming them for a small child, resulting in (rising)-falling intonation contours. Words and nonce-words were closely matched according to a number of acoustic parameters, i.e., duration of the target word, duration of the stressed syllable, mean f0 in stressed syllable, and f0 excursion of the accentual fall in the target (H^∗^ L-%), see Supplementary Table 3.

The words and nonce-words were also matched for speaker affect. To this end, all twelve words and nonce-words from the u-varied list were presented together with twelve less emphatic recordings of the same word and the same speaker (not used as experimental stimuli but recorded for the purpose of the rating task). Ten listeners rated these tokens (which occurred in both the u-varied and u-only condition) on a scale from 1 (= not enthusiastic at all) to 5 (= very enthusiastic). The words received an average rating of 3.92 (SD = 0.71), the nonce-words an average rating of 3.89 (SD = 0.76), corroborating that the words and nonce-words do not differ in perceived speaker affect.

##### Experimental Lists

The recordings of the experimental tokens were concatenated into four word lists and four nonce-word lists for both the u-varied and the u-only condition. The lists only differed in the order of tokens. Following van Heugten et al. (2018), the order of (nonce-)words varied within each list. Every token appeared only once. Moreover, no more than two monosyllabic or bisyllabic (nonce-)words occurred immediately adjacent. Across lists, each token appeared in early, mid and late positions of the list. Two of the four lists of each vowel-type were mirror lists of each other. Each list hence contained all twelve tokens (word or nonce-word tokens, respectively). The tokens were equated for loudness (65 dB) and concatenated with silent inter-stimulus intervals (ISIs) of approx. 750 ms, see van Heugten et al. (2018). To ensure an equal duration of all lists, the ISIs were adapted to reach list durations of 15 s (ISI was the same for each list and ranged from 742 to 756 ms). Each child received all four word lists and all four nonce-word lists of one vowel-type (vowel type was manipulated between-subjects). We constructed six different experimental randomizations of the above lists (i.e., of eight trials each), such that word lists and nonce-word lists did not appear more than two times in a row. Moreover, in all randomizations of the lists, word and nonce-word lists were balanced for experimental half, i.e., two of the four word lists and two of the four nonce-word lists occurred in one experimental half. Three of the six randomizations started with a word list, while three started with a nonce-word list. Randomizations were the same for u-varied and u-only conditions.

### Procedure

The remote familiar word paradigm consisted of eight trials in total (four word lists and four nonce-word trials, 15 s each). Each trial started with a colorful attention getter (taken from Frota et al., 2014), which was presented for 1 s (cf. Figure 2C). Then one of the word or nonce-word lists was played, accompanied by the visual presentation of a colored checkerboard. The sound played for the total duration of the list (and was hence not child-controlled). For each experiment version (u-only and u-varied), the six different randomizations of trials described above were distributed across participants. For the analysis of looking times, two of the trials of each child were coded manually on a frame-by-frame basis for looks in ELAN. The analysis was based on the automatic coding (cf. see section “Manual and Semi-Automatic Analysis of Looking Behavior”).

### Results

The looking times were slightly left-skewed, which is why we transformed them using a square-root transformation, see Eq. 1. The negative sign ensured that longer transformed looking times correspond to longer raw looking times.

(1) transformed_lookingtime=-sqrt⁢(16,500-lookingtime)

**FIGURE 4:**
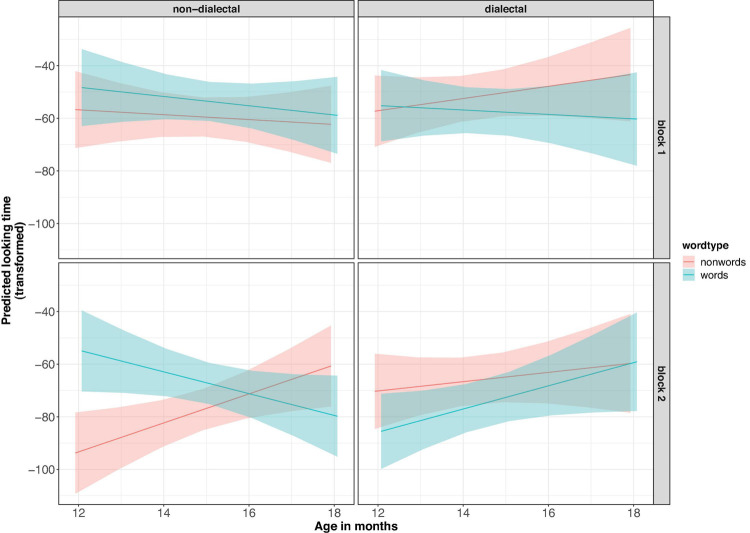
Predicted effects of the model in Experiment 1. The *y*-axis shows the transformed looking times – sqrt(16,500-lookingtime). Higher values indicate longer looking times.

The transformed looking times were analyzed in a linear mixed-effects regression model with *group* (non-dialectal vs. dialectal, treatment-coded), *word-type* (word vs. nonce-word, treatment-coded), *vowel-type* (u-only vs. u-varied, sum-coded) and the control predictors *block* (first vs. second block of trials, sum-coded) and scaled *age in months*. Block was included to test whether looking time differences are already present at the onset or develop over the course of the experiment, due to exposure to the stimuli (cf. analysis in Poltrock and Nazzi, 2015). Sum coding of predictors allowed us to focus on the main factors of interest, *group* and *word-type* in the summary()-tables. *Participants* were added as random effect (Baayen et al., 2008). Adding experimental list as random effect did not lead to model convergence. If the model converged, *block* and *vowel-type* were added as random slopes for participants. Due to convergence issues, only *block* was kept as random slope. The final model showed a significant four-way-interaction between *word-type*, *group*, *block* and *age* [*F*1_(1, 255)_ = 10.0, *p* < 0.005], see Figure 4 for marginal effects of the model. There was no effect of *vowel-type* and no interaction between *vowel-type* and any of the other factors (*p* > 0.1). A Bayes factor analysis (Morey and Rouder, 2018) showed that the model without *vowel-type* as predictor was more than 10,000 times more likely than the model with *vowel-type*. We investigated the four-way-interaction more closely by fitting separate models for the non-dialectal and dialectal groups.

**FIGURE 5:**
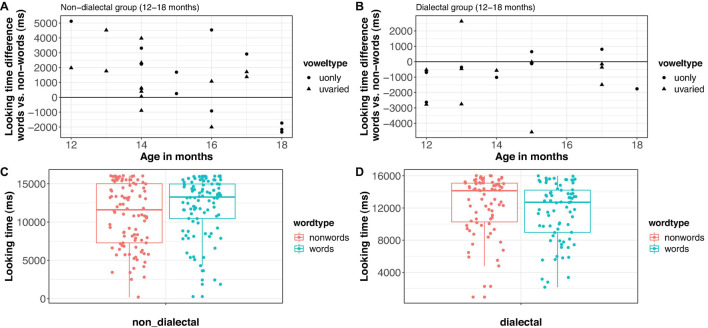
Looking time difference between words and nonce-words in Experiment 1 [**(A,B)**: each dot represents the average of one child] and looking times by word type [**(C,D)**: each dot represents one trial]. Left panels: non-dialectal children, right panels: dialectal children.

Figure 5 shows the raw looking time differences per child (panel A for non-dialectal, panel B for dialectal children) and the raw looking times per trial (panel C for non-dialectal, panel D for dialectal children). The non-dialectal group showed a main effect of *word-type* [*F*_(1, 146)_ = 7.4, *p* < 0.01], with longer looking times to word lists than nonce-word lists (ß = 7.9, SE = 2.9) and of *block* [*F*_(1, 23)_ = 19.9, *p* < 0.0005], with longer looking times in block 1 than block 2. The effect size (Hedge’s g) for the effect of *word-type* was 0.43, 95% CI [0.11;0.76] (small effect). Furthermore, there were significant interactions between *word-type* and *age* [*F*_(1, 146)_ = 10.2, *p* < 0.005], and between *word-type*, *age* and *block* [*F*_(1,146)_ = 7.2, *p* < 0.01], see left panel of Figure 4. The familiarity preference decreased with increasing age and was more pronounced in block 2, in particular for the younger children. Separate analyses by block showed longer looking times to words than nonce-words in block 1 [*F*_(1, 72)_ = 3, *p* = 0.08] and an interaction between *word-type* and *age* in block 2 [*F*_(1, 73)_ = 20.2, *p* < 0.0001], see Figure 4, left panel.

The dialectal group showed significant main effects of *word-type* [*F*_(1, 127)_ = 4.7, *p* < 0.05], with longer looking times to nonce-words than to words (ß = 7.1, SE = 3.3) and of *block* [*F*_(1,127)_ = 25.1, *p* < 0.001], with shorter looking times in block 2. Furthermore, there was a three-way-interaction between block, *vowel-type* and *age* [*F*_(1,127)_ = 6.3, *p* < 0.05]. The effect size (Hedge’s g) for *word-type* was 0.34, 95% CI [−0.64; −0.04] (small effect).

### Discussion

Experiment 1 showed looking time differences between word and nonce-word lists for both non-dialectal and dialectal children. However, the preference went in opposite directions: Non-dialectal children preferred words over nonce-words, while dialectal children preferred nonce-words over words. With regard to the non-dialectal children, who primarily receive Standard German input, we hence replicated the familiar word preference with German children in a home-setting using an App. We further showed that the familiar word preference was not affected by segmental variation of the stimuli (u-varied vs. u-only), but the familiarity preference was stronger in block 2, in particular for the younger children. The familiarity preference for words over nonce-words is in line with a number of studies that tested children in different languages in the lab (Swingley, 2005 for Dutch; Best et al., 2009 for American English; van Heugten and Johnson, 2014 for Canadian English; Poltrock and Nazzi, 2015 for French; Vihman and Majorano, 2017 for Italian, among others). At 18 months, the pattern seems to slowly develop into a novelty preference (Figure 5A). A similar decline in familiarity preference was also observed in Vihman et al. (2007). We will come back to this reversal of preferences in Experiment 2.

The dialectal children, who are exposed to more variability in word forms (both dialectal and Standard German word forms), showed a preference for the nonce-word lists, suggesting that they also recognized the Standard German word forms. The direction of the preference, however, is rare in the literature on this paradigm. To be more confident about the obtained effect in dialectal children, which was indeed unexpected, we challenged its stability by removing the child with a particularly large novelty preference (>4,000 ms); this looking time difference was 2.3 SD beyond the mean and does hence not qualify as an outlier in a strict sense. Moreover, we reran the analysis with an adapted looking time measure, excluding looks that occurred after a sequence of “no look” frames longer than 2 s (to simulate a child-controlled paradigm one would have used in the lab). This affected about half of the trials (*N* = 70, 46%), nevertheless the pattern of results did not change. In both cases, the novelty effect persisted, which corroborates its robustness.

Novelty preferences have been associated with more mature linguistic processing in the literature. DePaolis et al. (2016), for instance, showed that within one and the same age group (10-month-old children), successful recognition of familiar words can surface either as a familiarity preference or a novelty preference. Children who showed a preference (familiarity or novelty) in that study were lexically more advanced (as measured by standardized vocabulary assessments, CDI, MacArthur Communicative Developmental Inventory) than the children who did not show a preference (equal looking times to both stimuli types). The novelty preference in dialectal children in Experiment 1 may hence be tentatively interpreted as an effect of more mature linguistic processing. To test this hypothesis, we looked at a sample of older non-dialectal children, which are, naturally, more mature than the 12–18-month-olds, and for whom one may expect a similar novelty preference (cf. Thiessen et al., 2005 for a similar rationale in word segmentation)^6^. For the familiar word paradigm, there are only very few studies with children older than 19 months (Carbajal et al., 2021), who are typically tested with non-native accents or with specific populations (Best et al., 2009; van Heugten and Johnson, 2014; Kalashnikova et al., 2016). van Heugten and Johnson (2014) report an interaction between word type and age and suggest that “over time, infants start preferring to listen to known over nonsense words” (p. 344). Kalashnikova et al. (2016) tested a control group of 26-month-old children with familiar accents and showed a reduction of the familiar word preference for 26-month-old children.

## Experiment 2: Non-Dialectal 18–24-Month-Old Children

Experiment 2 follows up on the different directions of preferences observed in Experiment 1 (familiarity preference in non-dialectal vs. novelty preference in dialectal children). If the novelty preference is indeed indicative of more mature processing and if the familiar word paradigm is still a valid method for 18–24-month-old children, we would expect a change in the direction of the preference toward a novelty effect in non-dialectal children as they grow older.

### Methods

#### Participants

Twenty non-dialectal children between 18 and 24 months of age were included in the analysis (10 female and 10 male). Their mean age was 20.6 months (SD = 1.6 months). Ten children were tested with the u-varied lists, ten with the u-only lists. Their mean dialect score was 1.5 (SD = 0.4). The highest education of the first parent equaled a high school degree (for 13 children), vocational training (5 children), A-levels (1 child), and O-levels (1 child). Eight more children were tested, but not analyzed because the child did not complete the test (2 times), was not in the frame (2 times), was reported to be ill or impaired (3 times) or because of technical issues (1). This resulted in a dropout rate of 28.6%.

#### Materials and Procedure

The materials and the procedure were identical to Experiment 1.

### Results

**FIGURE 6:**
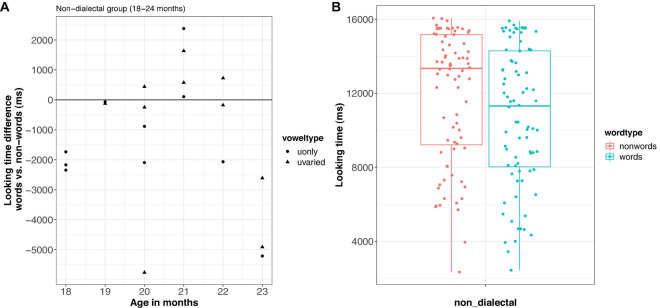
Looking time difference between words and nonce-words in Experiment 2 **(A)** and looking times by word type **(B)**.

The raw looking time differences per child and looking times per trial are shown in Figure 6. The looking times were transformed and analyzed as in Experiment 1. The final model showed significant effects of *word-type* [*F*_(1, 137)_ = 6.6, *p* < 0.05], *age* [*F*_(1, 17)_ = 8.4, *p* < 0.05], *vowel-type* [*F*_(1, 17)_ = 5.4, *p* < 0.05], and *block* [*F*_(1, 137)_ = 19.1, *p* < 0.001]. Furthermore, there was an interaction between *vowel-type* and *block* [*F*_(1, 137)_ = 4.9, *p* < 0.05]. Importantly, children looked longer to the nonce-word lists than to the word lists (ß = 9.1, SE = 3.5, Hedge’s *g* = 0.51, 95% CI [−0.96; −0.07], medium effect). Furthermore, children looked longer to the u-varied lists than to the u-only lists (ß = 21.1, SE = 6.7), especially in block 1. Looking times were furthermore shorter for older children (ß = −8.3, SE = 2.9) and in the second block (ß = −7.7, SE = 5.0).

**FIGURE 7:**
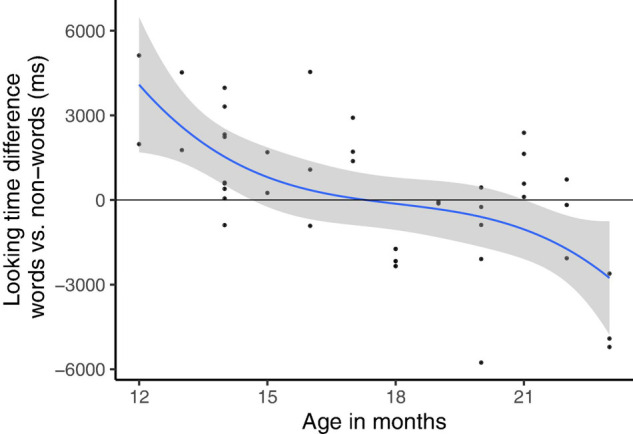
Development of looking time difference for non-dialectal children across age.

Figure 7 shows looking time differences for non-dialectal children across age in one figure. It demonstrates how the familiarity preference (overserved in Experiment 1) slowly develops into a novelty preference (Experiment 2) as a function of age.

### Discussion

In Experiment 2, non-dialectal 18–24-month-olds preferred nonce-words over words, suggesting that non-dialectal children indeed develop a novelty preference as they grow older, with longer looking times to nonce-word lists than to word lists. This tendency toward a novelty preference with increasing age has already been foreshadowed in non-dialectal children in Experiment 1: There, the familiarity effect was largest for the younger children in that group. It is not surprising that older children (18–24-months-olds) are developing a growing interest in nonce-words. At this age, the vocabulary develops very rapidly (e.g., Dapretto and Bjork, 2000) and an interest in novel words is the best way to further increase a child’s lexicon. Importantly, the looking behavior of older non-dialectal children resembles the behavior of the 12–18-month-old dialectal children who grow up with dialect forms in addition to Standard German (see Experiment 1). The fact that older non-dialectal and younger dialectal children both show a novelty preference does not necessarily mean that the cause is of the same origin. One generalization we can still infer from our findings is linguistic maturation (caused by a more variable input due to Standard German and dialectal forms in the dialectal group of Experiment 1 or caused by increased age in Experiment 2). We will further discuss this interpretation in the “General Discussion” section.

## General Discussion

The present paper tested word form recognition in German children aged 1–2 years, growing up with Standard German (non-dialectal children, mostly recruited in urban areas) or with Standard German *and* an additional dialect (dialectal children, mostly recruited in rural areas). Data collection was made possible by an App that allowed parents to run the experiment at home, using an experiment-controlled version of the familiar word paradigm. As predicted by H1, non-dialectal German 12–18-month-old children showed the preference for familiar over nonce-words established in the literature (cf. Carbajal et al., 2021). The familiarity preference was stronger for younger children, in particular in the second half of the experiment. From 18 months of age onward, this familiarity preference slowly developed into a novelty preference, with longer looking times for nonce-word than word lists. The task might have become successively simpler which increases the likelihood of a novelty preference and an increased interest in novel items. These results extend earlier findings on the familiar word preference for a different language (German) and for an older age group (12–18 months). Our main focus, however, lies on the dialectal German children, who receive a lot of variability in their daily input. The group of dialectal 12–18-month-olds showed a novelty preference comparable to older non-dialectal children. Since novelty preferences are rare within this paradigm, this finding was particularly unexpected (recall that based on the literature we had assumed a later occurrence of a familiarity preference for the dialectal group as compared to the non-dialectal group, cf. H2). We therefore critically assured that the novelty preference is indeed statistically robust. Finally, we concluded that this preference pattern is most likely to be attributed to more advanced linguistic skills in dialectal children, due to experience with variability in word forms. The segmental variability of the word (and nonce-word) lists did not affect the familiarity or novelty preferences. This suggests that specific alternations can be tested in the familiar word paradigm, which allows us to investigate the nature of early word representations with this paradigm (see section “Direction of the Preference”).

In the following, we first reflect on the classification into dialectal vs. non-dialectal children (see section “Operationalization of Dialectal vs. Non-dialectal”). Then, we discuss the direction of the preference (see section “Direction of the Preference”) before turning to the nature of lexical representations in dialectal children (see section “Effects of Dialectal Exposure on Lexical Development”). We conclude with an evaluation of the remote testing procedure using the App (see section “Evaluation of the Remote Testing Using the App”).

### Operationalization of Dialectal vs. Non-dialectal

In a first attempt to study the role of dialectal variability in children’s word form recognition, we used a binary classification procedure. This binary classification of children into a dialectal vs. non-dialectal group was primarily based on a perceptual measure, i.e., the impression of dialectal strength by a group of four coders. The coders came from different areas of Germany in order to avoid that familiarity with a particular dialect skewed the ratings in any form. Our coding system of dialect strength proved to be fairly reliable, which is in line with other studies that also reported high interrater reliability for the perceptual coding of dialect strength, even among lay coders (Ryan, 1973: Kendall’s *W* = 0.71; van Bezooijen and van Hout, 1985: ru = 0.93; Grondelaers et al., 2015: α = 0.6). This suggests that native speakers of a language are able to reliably perceive and indicate the strength of dialectal usage. Furthermore, previous studies have established that subjective measures of dialectal strength and objective phonetic measures are highly correlated (e.g., euclidian distance of first and second vowel formants from a reference, cf. Grondelaers et al., 2015), which further corroborates the validity of such coding systems.

In our study, the ratings reflect the perceived prevalence of dialectal deviations from expected Standard forms (recall that perfectly Standard German stimuli were coded as 1, stimuli with a few slight dialectal features as 2, with more dialectal features as 3, and 4 was used for highly dialectal productions). Although it is unlikely that the coders tracked these frequencies in an accurate one-to-one manner, phonetic transcriptions of the realizations of the subset of the recordings showed a very high correlation (Spearman’s rho > 0.88) between the perception of dialectal strength and the number of word forms that deviated from Standard German forms. Taken together, our system seems to have reliably grouped children into non-dialectal vs. dialectal children. Nevertheless, more fine-grained approaches are conceivable.

Our binary grouping aimed at serving as a first approximation toward operationalizing the dialect-induced variability in word forms, which allowed us to investigate the development of lexical representations. However, given that the use of dialect ranges over a continuum (Schwarz, 2014), and that present-day dialectal forms approach Standard German word forms (e.g., Kleber, 2020), it may be more valid to include dialectal strength as a continuous measure in the analyses for future studies, cf. Levy et al. (2019) for the role of experience in the processing of accented speech (unfamiliar regional and foreign) in 9-year-old children; and Porretta et al. (2016) on the influence of foreign accentedness and experience in adult word recognition. In addition, it may be helpful to include more questions on the frequency of dialect use and a self-assessed rating of dialect strength, similar to questionnaires used for bilingual children (e.g., Levy et al., 2019; DeCat, 2020).

### Direction of the Preference

The two groups tested in our study revealed effects of different directions. Almost all of the previous studies using the familiar word paradigm showed a familiarity preference [*N* = 18 out of 32, see lower half of Figure 2 in Carbajal et al. (2021)] if there was an effect, rather than a novelty preference. Most of the children in the familiar word paradigm were younger than 12 months of age (25 out of 32 studies in Carbajal et al., 2021). There is only one study that showed a novelty preference for linguistically more mature children (as measured by higher CD scores), cf. DePaolis et al. (2016). This study, along with previous reflections on the direction of effects (Hunter and Ames, 1988; Houston-Price and Nakai, 2004; Butler et al., 2011), led us to interpret the novelty preference in terms of linguistic maturation. In this section, we discuss the novelty preference in more detail.

We first compare the dialectal children to the non-dialectal children in our study and the results of these dialectal children to the behavior of bivarietal children studied by van Heugten and Johnson (2017). Recall that van Heugten and Johnson (2017) showed that Canadian English children exposed to multiple varieties of English (including foreign-accented speech) only recognized words at the age of 18 months, which is the upper age limit in the dialectal group in our study. There are a number of differences to our study that may have affected the seemingly contradicting direction of the effect, including (a) a remote experiment-controlled vs. a lab-based child-controlled procedure, and (b) the amount and kind of exposure to the variety tested in the paradigm and to other varieties. Regarding (a), the procedure may certainly influence the results, but it is hard to predict in which way. In a remote setting, there is probably more distraction, which may lead to smaller effect sizes, but hardly to a reversal of the effect. As discussed in section “Discussion,” a different analysis of the looking times, which is closer to a child-controlled procedure, did not change our pattern of results. Furthermore, all of our groups were tested with the App, but there were still differences in preferences. We hence conclude that factors beyond the mere difference in procedure need to account for the difference in findings. We see the most striking differences with regard to (b). However, the actual amount of exposure is difficult to compare. For the bivarietal children in van Heugten and Johnson (2017) Canadian English was available 34% of the time. We do not have such an estimate, but we know, from a subsample of the children, that Standard German word forms are frequent in the input of dialectal speech as well (amounting to 66% of the word forms). This difference in input frequency may explain why our dialectal children recognized the word forms earlier than the bivarietal Canadian children in van Heugten and Johnson (2017). Beyond the *amount* of input in the variety tested in the word form recognition paradigm [Canadian English in van Heugten and Johnson (2017) and Standard German in our study], which clearly differed in the two studies, the *kind* of input was also different: in van Heugten and Johnson (2017), only one caregiver had an accent different from the one tested in the experiment. It is likely that not many other people speak the same variety, so that exposure to this variety is limited to a single speaker. From the literature on word recognition and word learning, we know that variability (of different sorts) may have beneficial effects on the formation of lexical representations (Singh, 2008; Rost and McMurray, 2009; Höhle et al., 2020). In a study concerned with variability induced by different speakers, Rost and McMurray (2009), for instance, demonstrated that 14-month-old children benefited when novel objects were labeled by different speakers (as compared to single-speaker labeling), cf. Höhle et al. (2020). Singh (2008) compared whether or not stimuli in a familiarization phase showed variability (mixed affect) or not (only positive or negative affect). Their results similarly show that 7.5-month-old children form more specific lexical representations in the high- than in the low-variability condition. The benefits of high variability have so far been documented for variability in the experimental setting (e.g., habituation). In our case, however, speaker and dialectal variability is present in the daily, long-term input that a child receives. This might have indeed boosted the formation of lexical representations (or resulted in different kinds of representations), and in turn, might have very likely led to a novelty preference.

### Effects of Dialectal Exposure on Lexical Development

In this section, we briefly reflect on the nature of lexical (and prelexical) representations. While there are a number of studies that have investigated the nature of lexical representations in 20–24-month-old multivarietal children (Floccia et al., 2012; Durrant et al., 2015; van der Feest and Johnson, 2016), findings are inconclusive [e.g., Durrant et al. (2015) proposed underspecified representations because the children from the South-West of England recognized both correctly pronounced words and mispronunciations while Floccia et al. (2012) found that bivarietal children did not recognize the words when spoken in the non-rhotic variety of their parents, only in the rhotic variety of the community]. These studies are hard to compare, not least because of the variability in the language varieties, sound contrasts, conditions (same vs. different speaker) and age groups that were tested. The question about the nature of lexical representations in those children remains and goes back to the early stages of development that can be addressed with the familiar word paradigm. The finding that dialectal 12–18-month-old children exhibit looking time differences between Standard German word and nonce-word lists (longer looking times to nonce-word lists) suggests that they recognize Standard German word forms. This novelty preference is already present in the first half of the experiment, showing that dialectal children knew these Standard German word forms before the experiment started. Our results seem to rule out a single storage of the dialectal word form only [as suggested by Floccia et al. (2012) on the basis of referential word recognition studies]. The next step is thus to test whether dialectal children will exhibit a similar novelty effect as shown for Standard German stimuli when hearing stimuli spoken in their own dialect or in an unfamiliar dialect (with unattested sound alternations).

The novelty preference of dialectal children suggests that they have formed different representations than the non-dialectal children. Currently, we assume that dialectal children link the word forms of the two varieties to each other, either at the prelexical level, where, for example, [u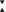] is linked to [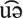], or at the conceptual level, where the concept FOOT is linked to [fu
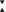
s] and [f
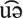
s]. This would allow them to recognize both Standard German and dialectal forms efficiently. This hypothesis is in line with e.g., Schmale et al. (2011), who showed that “exposure to phonetic variability [during word learning] leads to more robust representations by promoting broader lexical categories” (p. 1105). The additional connections may lead to the observed advantage in processing, exhibited as a novelty preference. There are other studies suggesting different processing mechanisms as a consequence of bilingual input (Meuter and Allport, 1999; Costa and Santesteban, 2004). These studies have shown asymmetric language switching costs in picture naming for L2 speakers, but symmetric switching costs for bilingual speakers. The prediction of more connected representations in dialectal children will be tested in future studies. If our assumption is correct, we predict a novelty preference for dialectal stimuli as well, while stimuli from unfamiliar dialects will not be recognized. For these studies it was relevant to test whether the effects of word type are independent of the segmental nature of the stimuli. In the present study, we compared u-only lists (in which all items contained the stressed vowel [u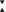]) to u-varied lists (in which half of the items contained [u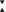] and half contained other vowels). Both types of lists resulted in the same familiarity (or novelty) preferences. These stimuli hence proved to be well-suited to investigate the nature of representations more closely.

Once more data will be collected with the App, it may also be interesting to differentiate between the dialects the children receive. Currently, most of the dialectal children grow up in the Alemannic dialect area and it is unclear how well the results generalize to other, more conservative, dialects with less transparent phonological mappings between Standard German and dialectal form.

### Evaluation of the Remote Testing Using the App

Using the App had a number of advantages compared to traditional laboratory settings: First and foremost, we had access to a larger, more diverse group of participants, in particular from rural areas in which children are exposed to more dialectal forms than the typical child participant tested in the lab. It has to be noted, however, that access to these communities was often only possible through personal contacts who then encouraged their network(s) to participate. This sometimes resulted in participation of children who did not fall into our primary group of interest, given the hypotheses (e.g., older children). Ads in local newspapers and flyers in kindergartens proved to be inefficient for recruitment purposes. Another advantage of the App is that parents do not have to make an appointment to come to the lab. They can freely choose to start the experiment whenever the child is in a good mood. The child likewise benefits from a familiar environment (in contrast to potentially intimidating settings in the lab). Finally, the time investment for most of the drop-outs was minimal, because most of the exclusion criteria were extractable within minutes. The automatic coding of the looking behavior worked extremely well, with a high level of accuracy. The most time-consuming aspect was the manual coding of two of the trials, which took 6–10 min for trained annotators (for one trial of 15 s duration). The manual rating of the dialectal input was done in less than 2 min.

However, home testing leaves researchers with less control over environmental factors. There were cases in which disruptions occurred during testing (e.g., by people talking in the background), which would not have occurred in the lab. Furthermore, the home set-up also assigned more responsibility to the parents, e.g., in reading the instructions, placing the iPad at an appropriate distance and angle, and the restriction not to interfere. Even though parents were explicitly told to participate only once with their child, some parents initiated several attempts (in these cases, we analyzed only the first attempt). The dropout-rate was higher for the older age group (28.6%) than for the younger age group (13.7%); on average it was 19.0% and thus comparable to familiar word recognition studies tested under laboratory conditions (on average 16% in the papers included in Carbajal et al., 2021). iPad-specific reasons for drop-outs were an inadequate position of the tablet so that the child was not in frame, technical issues or loud background noise, but these occurred only very rarely (6 out of 15 dropouts). Furthermore, the experiment-controlled duration of the trials led to boredom in some children (and to ceiling effects in others), which could have been avoided with a child-controlled set-up. However, a child-controlled set-up requires online-coding of looking times in order to stop trials and initiate new ones. This technical solution was not available at the time of testing. An additional aspect, which typically plays a minor role in the lab, concerned data protection: Many parents were worried about uploading the video data, which prevented some families from participation. Other parents did not have access to iPads, which we solved by lending iPads from the lab to interested parents. Finally, in an attempt to make the use of the App more attractive and to prevent parents from exiting the App before starting the word recognition experiment, the background questionnaires were shorter than those used in the laboratory. This compromise, however, made it hard to capture the input that children received based on the questionnaire alone (e.g., some parents mentioned two languages under the first language field of the App (which we interpreted as bilingual), others mentioned further languages in subsequent language fields of the App). Since we did not ask them to specify in which situations, how often and by whom other languages than German are used, it was hard to specify exclusion criteria. The recordings of two caregivers (which we got from four children) definitely helped to get a better understanding of the child’s linguistic environment, but only few parents made use of this option. We do not know whether making this part of the experiment compulsory would prevent them from taking part.

The effect sizes in our App-based experiments were all lower than the average effect size reported in child-controlled familiar word paradigms tested in the lab. Although lower effect sizes may have a number of different causes, we assume that the remote testing with the experiment-controlled stimulus duration may be relevant. Replicating our study with the same group of children and stimuli in the lab will shed light on this issue. In any case, the data suggests that the familiar word effect is robust enough to be replicated with experiment-controlled stimulus duration in home environments. As discussed above (see section “Direction of the Preference”), the reversed effect directions observed across groups is very unlikely to be due to App-based testing, and builds on group differences instead; otherwise, we would have observed a similar pattern in both groups of children.

In future research, a browser version of the experiment could help making the study more widely accessible, even though this might come along with compatibility issues of individual browsers. Furthermore, we plan to test whether the manual coding effort can be further reduced with equally reliable results for the automatic coding. Finally, we are currently testing phonetic fingerprints to distinguish the non-dialectal from the dialectal children (Behrens-Zemek and Braun, 2021).

Home-based testing seems to be a viable option to gather looking-time data of children who grow up with a dialect, which allows us to investigate the development of word forms in populations that hear both Standard German and dialectal forms. The looking-time data indicates that Standard German word forms are recognized by dialectal 12–18-month-old children; the reversal of the preference (a novelty preference in dialectal as compared to a familiarity preference for non-dialectal children) suggests differences in word form representations, which will have to be investigated in more detail in future studies.

## Data Availability Statement

The stimuli and the raw data supporting the conclusion of this article are available on https://data.mendeley.com/datasets/gf7hsh932v/2.

## Ethics Statement

The studies involving human participants were reviewed and approved by University of Konstanz IRB Board. Written informed consent to participate in this study was provided by the participants’ legal guardian/next of kin. Written informed consent was obtained from the individual(s), and minor(s)’ legal guardian/next of kin, for the publication of any potentially identifiable images or data included in this article.

## Author Contributions

BB and KZ-R developed the idea and the design of the study and materials (in collaboration with JR), and the video coding protocol (in collaboration with NC and JK). KZ-R and JR recorded and prepared the experimental stimuli. NC, JK, and KZ-R trained and supervised the annotators for video coding. BB led the statistical analysis and drafted the manuscript. CZ developed the App. JP and BG contributed to the algorithms for automatic video coding. All authors wrote parts and edited the draft.

## Conflict of Interest

The authors declare that the research was conducted in the absence of any commercial or financial relationships that could be construed as a potential conflict of interest.

## Publisher’s Note

All claims expressed in this article are solely those of the authors and do not necessarily represent those of their affiliated organizations, or those of the publisher, the editors and the reviewers. Any product that may be evaluated in this article, or claim that may be made by its manufacturer, is not guaranteed or endorsed by the publisher.

## Footnotes

1
https://apps.apple.com/de/app/bsl-wortformerkennung/id1508534681

2
https://4dface.io/

3
http://wordbank.stanford.edu/

4
https://calculator.ku.edu/phonotactic/English/words

5 One reviewer pointed out that some of the nonce-words may be perceived as a mispronunciation of an existing word. In the literature, children from 11 months onward do not recognize words in which individual sounds (or features) are changed, especially for consonants (e.g., Swingley, 2005; Poltrock and Nazzi, 2015). To test whether the nonce-words may be understood as existing German words, we played all nonce-words to a group of eight student assistants. They were given 2 s to name a word that spontaneously came to their mind or to respond with “X” if no word occurred to them. Only four nonce-words led to associations with an existing word, whereby more than half of the student assistants, respectively, mentioned the same word. Overall, we find this proportion (or amount of deviance between words and nonce-words) to be comparable to other studies using the familiar word paradigm (Vihman et al., 2004; Swingley, 2005; van Heugten and Johnson, 2014; Vihman and Majorano, 2017).

6 There were not enough data points of dialectal 18–24-month-olds for comparison, unfortunately.
