# Supplementary material for: Remote Testing of the Familiar Word Effect With Non-dialectal and Dialectal German-Learning 1–2-Year-Olds
Source: Front Psychol. 2021 Dec 2;12:714363. doi: 10.3389/fpsyg.2021.714363 (PMC8674187; doi:10.3389/fpsyg.2021.714363)
Supplement: Supplementary file 1 [file Data_Sheet_1.docx]

Supplementary Material

# Supplementary Figures and Tables

| Standard | Intended form (IPA) | Dialectal form (IPA) | English translation |
| --- | --- | --- | --- |
| Geht die auch noch zum Schaf hin? | [geːt diː a͡ʊx nɔx t͡sʊm  ʃaːf hɪn] | [gɔːt dɪ a͡ʊ nɔ t͡sm̩ˈ ʃɛːflə nɑ] | Does she also go to the sheep? |
| Wo sie gegessen haben | [voː zi ɡəˈɡɛsən ˈhabən] | [voː sə ˈɡɛsə hant] | Where they ate |
| Sagst du | [zaːɡst dʊ] | [sa͡ɪʃ dʊ] | You say |

Table 1. Comparison of standard forms (second column) to dialectal forms (third column), together with the English translation (last column).


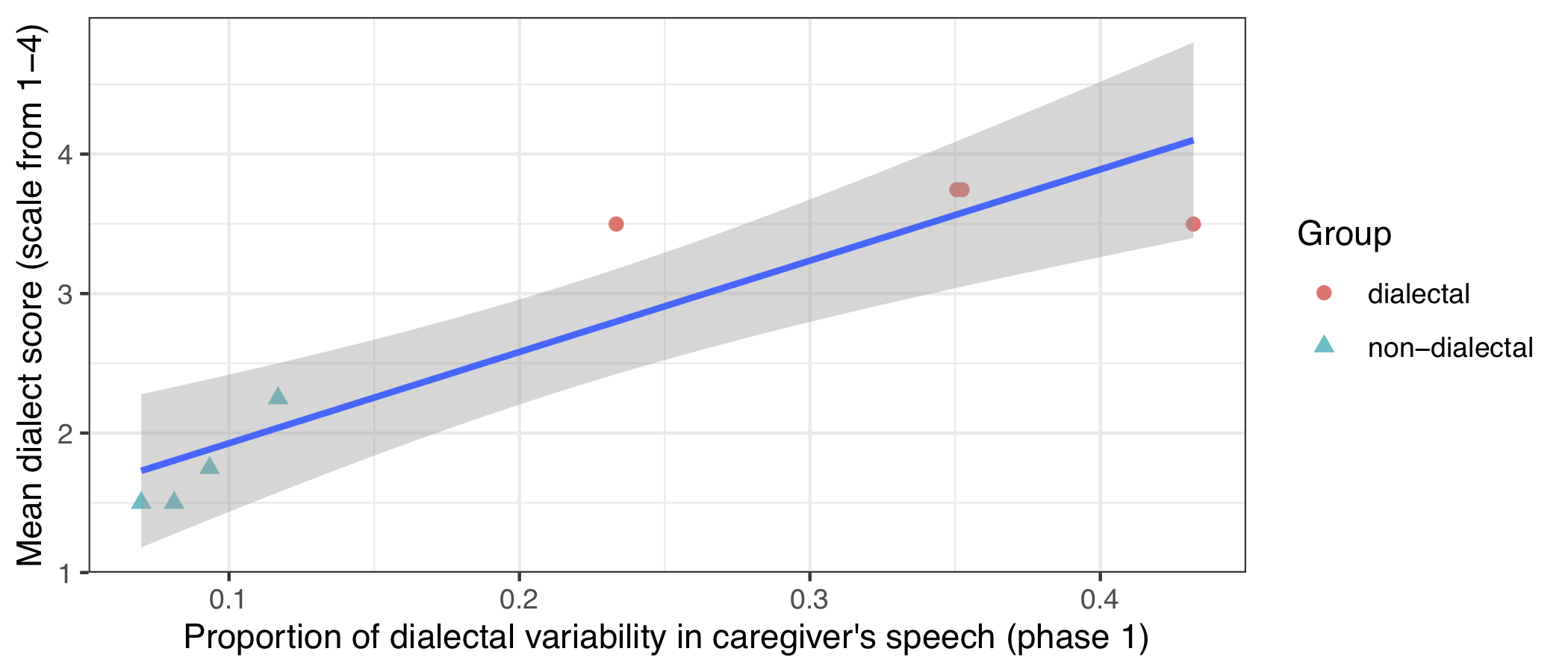


Figure 1: Correlation between perceived dialect strength (1-4) and proportion of dialectal deviations in the input.

|  | MannLog | WB_18m | WB_24m |
| --- | --- | --- | --- |
| u-only | 2.00 (0.85) | 0.24 (0.13) | 0.66 (0.18) |
| u-varied | 1.53 (0.52) | 0.30 (0.17) | 0.75 (0.09) |
|  |  |  |  |

Table 2. Average (and standard deviation) of frequency counts (MannLog) and production frequency at 18 (WB_18m) and 24 months (WB_24m) of age of u-only and u-varied word lists.

| **u-only** | Words | Nonce-words |
| --- | --- | --- |
| Duration of target (ms) | 596.3 (108.8) | 600.8 (108.9) |
| Duration of str. syllable (ms) | 450.5 (158.6) | 438.4 (184.1) |
| Mean f0 in str. syllable (Hz) | 282.3 (37.4) | 283.3 (36.3) |
| F0 excursion of fall (st) | 11.6 (1.0) | 12.0 (1.5) |

| **u-varied** | Words | Nonce-words |
| --- | --- | --- |
| Duration of target (ms) | 602.6 (122.7) | 608.0 (92.2) |
| Duration of str. syllable (ms) | 439.9 (176) | 443.2 (221.4) |
| Mean f0 in str. syllable (Hz) | 273.4 (40.9) | 275.1 (40.1) |
| F0 excursion of fall (st) | 11.9 (0.9) | 12.2 (1.2) |

Table 3. Acoustic parameters (mean values (and standard deviations)) of words and nonce-words.

**
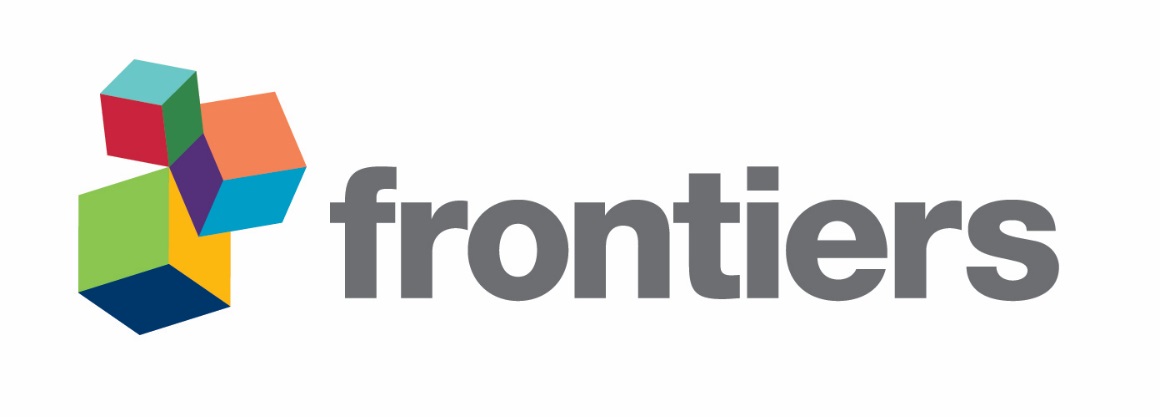
**
